# Synergistic Effect of Combinatorial Treatment with Curcumin and Mitomycin C on the Induction of Apoptosis of Breast Cancer Cells: A cDNA Microarray Analysis

**DOI:** 10.3390/ijms150916284

**Published:** 2014-09-15

**Authors:** Qian-Mei Zhou, Qi-Long Chen, Jia Du, Xiu-Feng Wang, Yi-Yu Lu, Hui Zhang, Shi-Bing Su

**Affiliations:** 1 Research Center for Traditional Chinese Medicine Complexity System, Shanghai University of Traditional Chinese Medicine, Shanghai 201203, China; E-Mails: tazhou@163.com (Q.-M.Z.); cqlw1975@126.com (Q.-L.C.); sbwsyt625@163.com (J.D.); xiufengw@sina.com (X.-F.W.); ava0048@163.com (Y.-Y.L.); zhanghuiman@126.com (H.Z.)

**Keywords:** curcumin, mitomycin C, synergistic effect, gene expression profile, breast cancer

## Abstract

In order to explore the synergistic mechanisms of combinatorial treatment using curcumin and mitomycin C (MMC) for breast cancer, MCF-7 breast cancer xenografts were conducted to observe the synergistic effect of combinatorial treatment using curcumin and MMC at various dosages. The synergistic mechanisms of combinatorial treatment using curcumin and MMC on the inhibition of tumor growth were explored by differential gene expression profile, gene ontology (GO), ingenuity pathway analysis (IPA) and Signal–Net network analysis. The expression levels of selected genes identified by cDNA microarray expression profiling were validated by quantitative RT-PCR (qRT-PCR) and Western blot analysis. Effect of combinatorial treatment on the inhibition of cell growth was observed by MTT assay. Apoptosis was detected by flow cytometric analysis and Hoechst 33258 staining. The combinatorial treatment of 100 mg/kg curcumin and 1.5 mg/kg MMC revealed synergistic inhibition on tumor growth. Among 1501 differentially expressed genes, the expression of 25 genes exhibited an obvious change and a significant difference in 27 signal pathways was observed (*p* < 0.05). In addition, Mapk1 (ERK) and Mapk14 (MAPK p38) had more cross-interactions with other genes and revealed an increase in expression by 8.14- and 11.84-fold, respectively during the combinatorial treatment by curcumin and MMC when compared with the control. Moreover, curcumin can synergistically improve tumoricidal effect of MMC in another human breast cancer MDA-MB-231 cells. Apoptosis was significantly induced by the combinatorial treatment (*p* < 0.05) and significantly inhibited by ERK inhibitor (PD98059) in MCF-7 cells (*p* < 0.05). The synergistic effect of combinatorial treatment by curcumin and MMC on the induction of apoptosis in breast cancer cells may be via the ERK pathway.

## 1. Introduction

Drug discovery has focused on the identification of the agents that can modulate individual target. Although some new drugs with an individual target have been discovered, limited treatment efficacy, poor safety and resistance profiles are often observed [1,2]. Due to these issues, the approach to drug design has been confirmed by successful clinical studies with multicomponent therapies [3,4], and efforts have been made for the discovery of combinatorial therapies using new drugs [5,6], and for the achievement of different synergistic effects.

When two drugs produce the same broad therapeutic effect, their combinatorial application also can produce the same effect to some extents, as compared with the summed effects of the individual drugs. The combination is pharmacodynamically synergistic if the effect is greater than the summed effects of the partner drugs [7]. Synergistic effects of drug combinations have been explored to achieve one or more favorable outcomes, such as enhanced treatment efficacy and decreased dosage at an equal or increased efficacy [8].

In addition, the combinatorial treatment efficacy of these substances depends on drug–drug interactions. The activity of curcumin is evaluated to compromise the pro-apoptotic activity of camptothecin, alkylating agents and anthracyclines [9]. On the contrary, curcumin reveals synergistic inhibition on cell growth and stimulation of apoptosis in human colon cancer cells when combined with chemotherapeutic drugs, such as bortezomib and 5-fluorouracil [10]. We have carried out anti-cancer studies in breast cancer cells through xenografts by using combinatorial treatment of curcumin and mitomycin C (MMC) [11,12,13]. However, the synergistic mechanisms of combinatorial treatment by curcumin and MMC on the inhibition of tumor growth are still unclear.

Tumor growth is not only due to the uncontrolled proliferation, but also due to the reduced apoptosis [14]. Therefore, the induction of apoptosis has become an effective strategy of cancer therapy. Additionally, recent reports have indicated that apoptosis is the activation of caspases by a signal transduction cascade [15]. Mitogen-activated protein kinase (MAPK) pathways have been implicated in the response to chemotherapeutic drugs [16]. MAP kinases regulate many cellular activities from gene expression to mitosis, movement, metabolism, and apoptosis [17]. Similarly, some responsiveness was regulated by ERK [18] and p38 [19] in breast cancer cells. It is of interest, therefore, to determine whether apoptosis is induced by the combinatorial treatment by curcumin and MMC through the ERK signaling pathway.

In the present study, the combinatorial treatment of curcumin and MMC-induced inhibition of tumor growth revealed synergistic effect in breast cancer xenografts. The ERK pathway was identified as a critical mechanism during the combinatorial treatment for the inhibition of tumor growth.

## 2. Results and Discussion

### 2.1. Effect of Combinatorial Treatment Using Curcumin and Mitomycin C (MMC) on Tumor Growth

**Figure 1:**
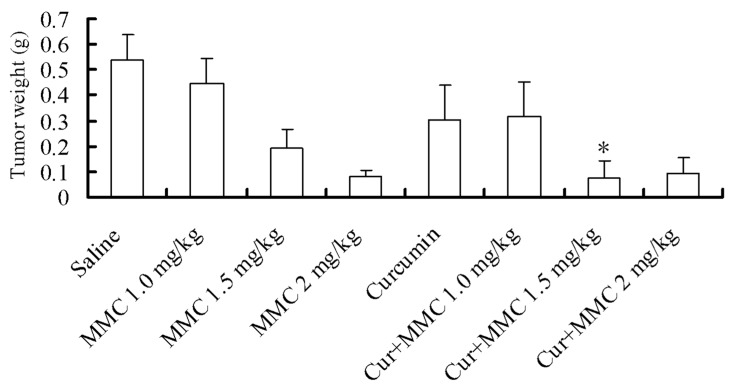
Effect of mitomycin C (MMC) or MMC combined with curcumin on tumor outgrowth in MCF-7 xenografts. Mice were first injected with MCF-7 cells for two weeks to establish xenograft. Mice were then administrated with MMC at the doses of 1.0, 1.5 and 2 mg/kg with or without curcumin. After four weeks treatments, the mice were sacrificed, and tumors were excised and weighed. * *p* < 0.05 *versus* 2 mg/kg MMC alone.

In order to determine the effect of curcumin/MMC on tumor growth, we established MCF-7 breast cancer xenografts. MMC treatment alone at the dose of 1.0 mg/kg had no significant effect on tumor growth (Figure 1). The weight of tumors was reduced by 44.21%, 64.53% and 84.52% during the treatment with 100 mg/kg curcumin, 1.5 and 2 mg/kg MMC alone, respectively. While, the side effect of 2 mg/kg MMC alone was more serious than other treatments (data not shown). The combinatorial treatment with curcumin and MMC at the doses of 1.0, 1.5, and 2 mg/kg, however, resulted in a more loss of tumor weight. The inhibitory rates were 40.91%, 86.51%, and 82.72%. Curcumin significantly enhanced the capability of MMC to reduce tumor mass (64.53% to 86.51%) when MMC was administrated at the dose of 1.5 mg/kg. Therefore, the combinatorial application of curcumin and MMC displayed a synergistic anti-tumor effect.

### 2.2. Gene Expression Profiles by cDNA Microarray Analysis

**Figure 2:**
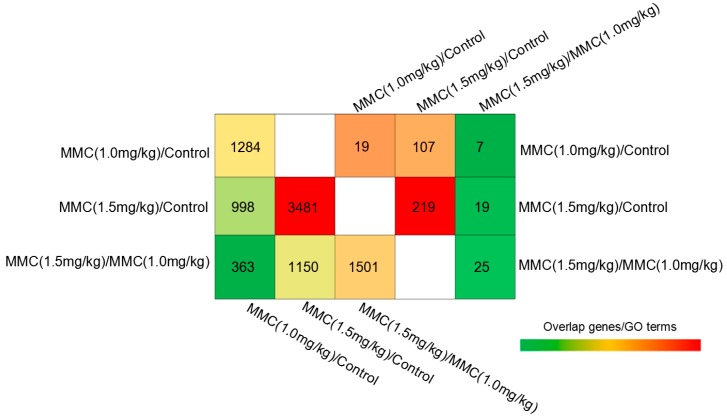
Venn diagram summarizing differentially expressed (DE) genes and gene ontology (GO) in tumor tissues with different treatments. Mice were treated as in Figure 1. Changes in gene expression were determined by cDNA microarray gene profiling. Genes with a false discovery rate (FDR)-adjusted *p* value (*p* < 0.05) and fold change larger than 1.5 were considered as DE genes. Above diagonal-DE genes; below diagonal-GO terms.

In order to establish the gene expression profile in tumors, the relative expression levels of mRNAs were analyzed with random module *t*-test of R package for exploring the significantly differential expression of mRNAs among tumor tissues subjected to curcumin/MMC treatment. As shown in Figure 2, we found 1501 differentially expressed mRNA in curcumin/MMC 1.5 mg/kg *versus* curcumin/MMC 1.0 mg/kg, 3481 mRNAs in curcumin/MMC 1.5 mg/kg *versus* control, and 1284 mRNAs in curcumin/MMC 1.0 mg/kg *versus* control. Furthermore, the number of overlapping genes among the curcumin/MMC 1.5 mg/kg, curcumin/MMC 1.0 mg/kg and control samples are shown.

In order to understand these DE genes holistically, we conducted functional enrichment analysis for DE genes using IPA analysis. Significant analysis was determined when *p* values were corrected for false discovery rate (FDR). Gene sets containing less than five gene overlapping were removed from IPA analysis. In our analysis, GO terms and pathways with an FDR-adjusted *p* value of less than 0.05 were retained. The number of representative GO terms of DE genes in each comparison is shown in Figure 2. Through GO analysis, 25 GO terms were observed in curcumin/MMC at the dose of 1.5 mg/kg *versus* curcumin/MMC at the dose of 1.0 mg/kg, 219 GO terms were observed in curcumin/MMC at the dose of 1.5 mg/kg *versus* the control, and 119 GO terms were observed in curcumin/MMC at the dose of 1.0 mg/kg *versus* the control. In addition, 19 GO terms were overlapped between curcumin/MMC at the dose of 1.5 mg/kg *versus* curcumin/MMC at the dose of 1.0 mg/kg and curcumin/MMC at the dose of 1.5 mg/kg *versus* control, 7 GO terms were overlapped between curcumin/MMC at the dose of 1.5 mg/kg *versus* curcumin/MMC at the dose of 1.0 mg/kg, and curcumin/MMC at the dose of 1.0 mg/kg *versus* the control, and 107 GO terms were overlapped between curcumin/MMC at the dose of 1.5 mg/kg *versus* the control and curcumin/MMC at the dose of 1.0 mg/kg *versus* the control (Figure 2). The variety of this phenomenon suggested that the different treatments using curcumin/MMC in tumor tissues likely had different molecular mechanisms.

### 2.3. Modulation of Signaling Pathways Following Combinatorial Treatment of Curcumin/MMC

**Figure 3:**
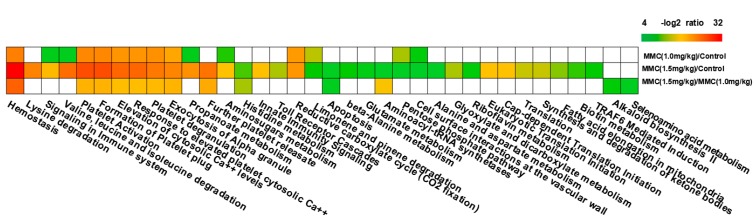
Significant pathways at the nominal level of 0.05 during the unpaired Student’s *t*-test. The DE genes in tumors treated with curcumin/MMC at the dose of 1.5 mg/kg and curcumin/MMC at the dose of 1.0 mg/kg were mapped to the IPA-defined work. The significance *p* values that determine the probability associated with the genes in the dataset and the canonical pathway alone were calculated by Fisher’s exact test, and are expressed as −log (*p* value). Green to red represent −log_2_ ratio from small to large.

Genes with significant change in expression following combinatorial treatment of curcumin/MMC at the dose of 1.5 mg/kg and curcumin/MMC at the dose of 1.0 mg/kg were assigned to different signaling pathways and subjected to ingenuity pathway analysis (IPA). Results showed that 88 pathways were differentially changed, which included 27 significantly altered pathways with −log (*p* value) ranging from 3.032 to 14.881. All of them are involved in apoptosis, cell cycle and mitosis (Figure 3). However, among these pathways, the −log (*p* value) of cell cycle was 3.032 and the ratio was 212 (data not shown). Therefore, these genes with more correlation with other genes that are necessary for apoptosis and cell cycle progression were selected (Figure 3).

### 2.4. Relationships among Genes by Signal–Net Analysis

**Figure 4:**
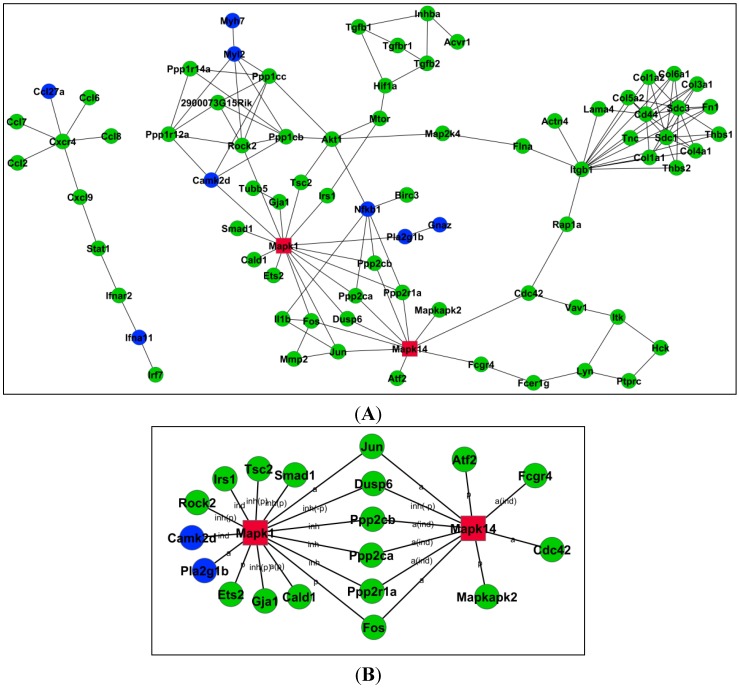
Signal–Net analysis of DE genes in significant pathways. The circles represent genes (blue, down-regulated; other colors, up-regulated. Mapk1 and Mapk14 associated with apoptosis in red). The size of the area represents the degree. (**A**) The differentially expressed mRNAs between curcumin/MMC at the doses of 1.5 and 1.0 mg/kg was observed. Gene regulatory networks based on Signal–Net database was established; (**B**) Apoptosis-related genes such as Mapk1 and Mapk14 were included. The relationship among of apoptosis-related genes was mapped.

Following the differentially expressed mRNAs between the curcumin/MMC at the dose of 1.5 and 1.0 mg/kg, we established gene regulatory networks based on Signal–Net database. The representative network is shown in Figure 4A. The network is highly weighted to evaluate the hub nodes (mRNAs) in terms of topological structure measurements. In the work, a hub node is defined to have more than five interactions in those stage-specific networks, and these hub nodes were highly affect the network architecture, thus implicating that the interesting potential modules were existed in the networks as the molecular pathways in biological system.

In addition, Betweenness Centrality represents the modulation capability of a gene, and Degree represents gene–gene interaction number. Indegree and Outdegree represent the upstream and downstream functions in the signaling pathway [20]. Key genes with Degree larger than five are listed in Table 1, which include apoptosis-related genes such as Mapk1 [21], Mapk14 [22], Rock2 [23], Akt1 [24] and Jun [25]. The relationship among these apoptosis-related genes was shown in Figure 4B.

### 2.5. Validation of DE Genes by Quantitative RT-PCR (qRT-PCR)

From Signal–Net analysis, two genes such as Mapk1 (ERK) and Mapk14 (MAPK p38) had more interaction with other genes, and were selected for further analysis. In order to determine the robustness of cDNA microarray gene expression profiling following the combinatorial treatment, quantitative RT-PCR (qRT-PCR) was employed to determine the change in the expression of the selected genes from Signal–Net analysis.

**Figure 5:**
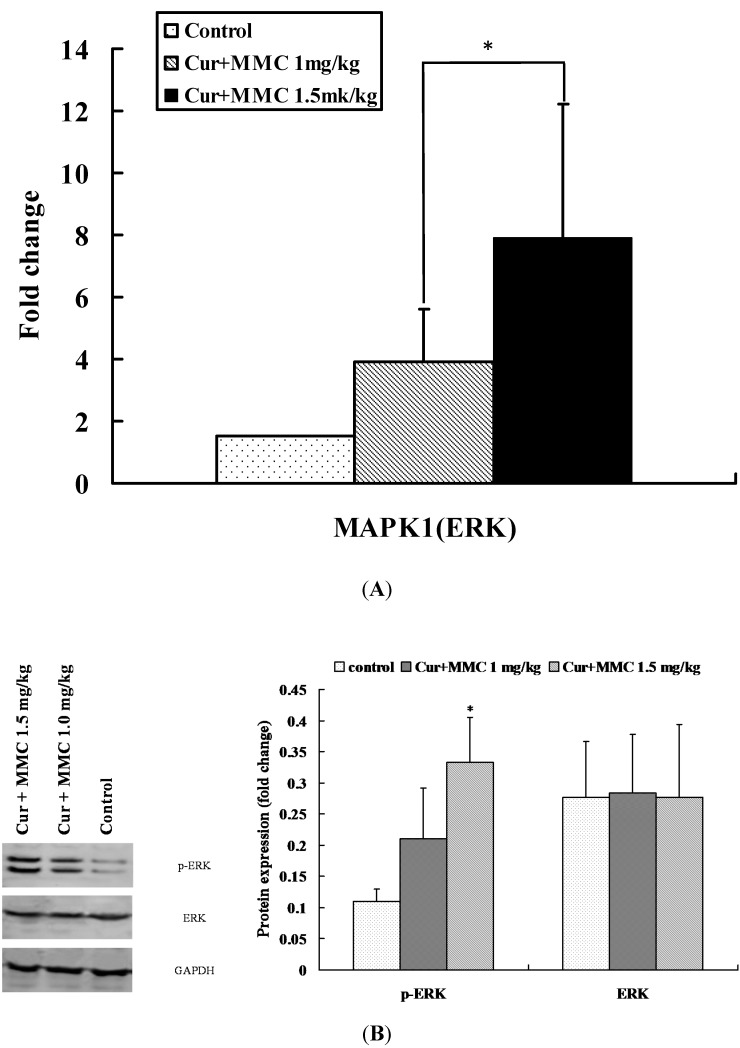
DE genes validated by quantitative RT-PCR (qRT-PCR) and Western blot analysis. (**A**) Total RNA was extracted from tumor tissues and qRT-PCR was performed as the description in Materials and Methods. Data represent as Mean ± SD from 4 determinations, and GAPDH was used to normalize the relative mRNA level. * *p* < 0.05; (**B**) Tumor lysates were analyzed for p-ERK and ERK with the corresponding antibodies. Values are mean ± SE from three independent experiments. * *p* < 0.05 curcumin plus MMC 1.5 mg/kg *versus* control.

The expression of ERK was increased by 3.74- and 8.14-fold in combinatorial treatment with curcumin/MMC at the dose of 1.0 mg/kg and curcumin/MMC at the dose of 1.5 mg/kg when compared with the control. Moreover, a significant difference between curcumin/MMC at the dose of 1.0 mg/kg and curcumin/MMC at the dose of 1.5 mg/kg was observed (*p* < 0.05). The expression of MAPK p38 had been observed in previous studies [12]. The expression of ERK by qRT-PCR fitted the pattern of cDNA microarray analysis well (Figure 5A). We next analyzed the expression of p-ERK and ERK in tumor grafts from the mice receiving either saline or drugs. The treatment of curcumin combined with MMC revealed an increase in the expression of p-ERK (Figure 5B). The results indicated that the change in gene expression following the combinatorial treatment with curcumin/MMC at the dose of 1.5 mg/kg and curcumin/MMC at the dose of 1.0 mg/kg was determined by microarray analysis, and confirmed by qRT-PCR and Western blot.

**Table 1:** Key genes (Degree ≥ 5) of Signal–Net analysis.

Gene Symbol	Betweenness Centrality	Degree	Indegree	Outdegree	Style
*Mapk1*	3.43 × 10^−2^	20	5	15	up
*Rock2*	1.17 × 10^−2^	13	1	12	up
*Mpak14*	2.25 × 10^−2^	11	3	8	up
*Akt1*	2.70 × 10^−3^	7	1	6	up
*Nfkb1*	2.27 × 10^−3^	6	1	5	down
*Mtor*	4.66 × 10^−4^	5	1	4	up
*Ppp1cb*	2.31 × 10^−3^	7	4	3	up
*Ppp1cc*	2.31 × 10^−3^	7	4	3	up
*Ppp1r12a*	1.49 × 10^−3^	6	3	3	up
*Itgb1*	3.91 × 10^−3^	14	14	2	up
*Fos*	2.10 × 10^−3^	6	4	2	up
*Jun*	2.10 × 10^−3^	6	4	2	up
*Myl2*	2.24 × 10^−3^	7	7	1	up
*Sdc1*	0	10	10	0	up
*Sdc3*	0	10	10	0	up
*Cd44*	0	8	8	0	up
*2900073G15Rik*	0	6	6	0	up
*Cxcr4*	0	6	6	0	up

### 2.6. Curcumin Significantly Improves the Tumoricidal Effect of MMC in MDA-MB-231 Cells

**Figure 6:**
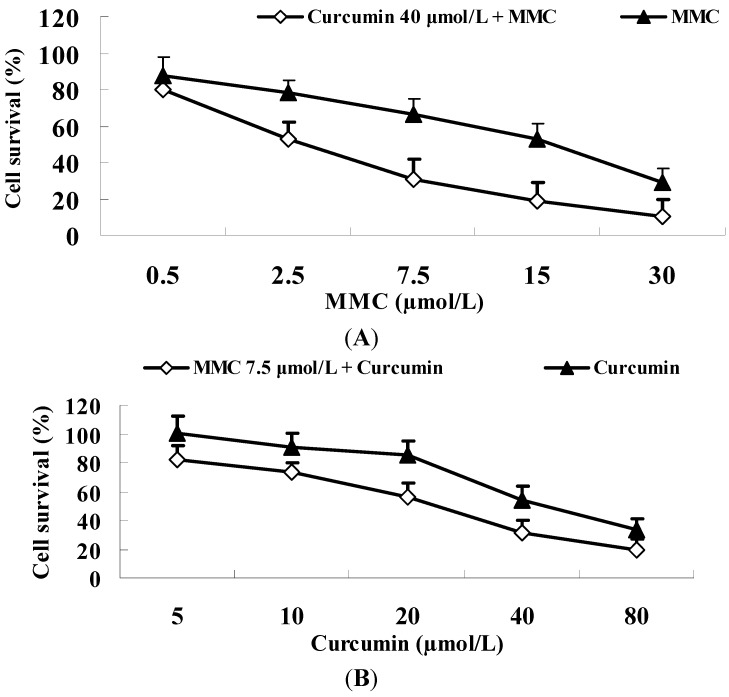
Sensitivity of breast cancer MDA-MB-231 cells to MMC and MMC plus curcumin. MTT assay was performed to determine cell number as described in Material and Methods. (**A**) MDA-MB-231 cells were treated with 0.5, 2.5, 7.5, 15 and 30 µmol/L MMC with or without 40 µmol/L curcumin for 48 h; (**B**) MDA-MB-231 cells were incubated in 5, 10, 20, 40 and 80 µmol/L curcumin with or without 7.5 µmol/L MMC for 48 h. Values are mean ± SE from three independent experiments.

It has shown that curcumin synergistically enhanced the anti-tumor capability of MMC in breast cancer MCF-7 cells. In order to test this hypothesis, we initially determined the cytotoxicity effect of MMC with or without curcumin on MDA-MB-231 cells. Cells were exposed to various MMC concentrations in the presence or absence of 50% inhibition (IC_50_) of curcumin (40 µmol/L) for 48 h (Figure 6). MMC treatment alone led to IC_50_ in cell number at 15 µmol/L to MDA-MB-231 cells. When MMC was combined with 40 µmol/L of curcumin, IC_50_ of MMC was reduced to 2.5 µmol/L in MDA-MB-231 cells, representing a 600% improvement in IC_50_ of MMC.

### 2.7. Apoptosis via ERK Pathway in MCF-7 Cells

**Figure 7:**
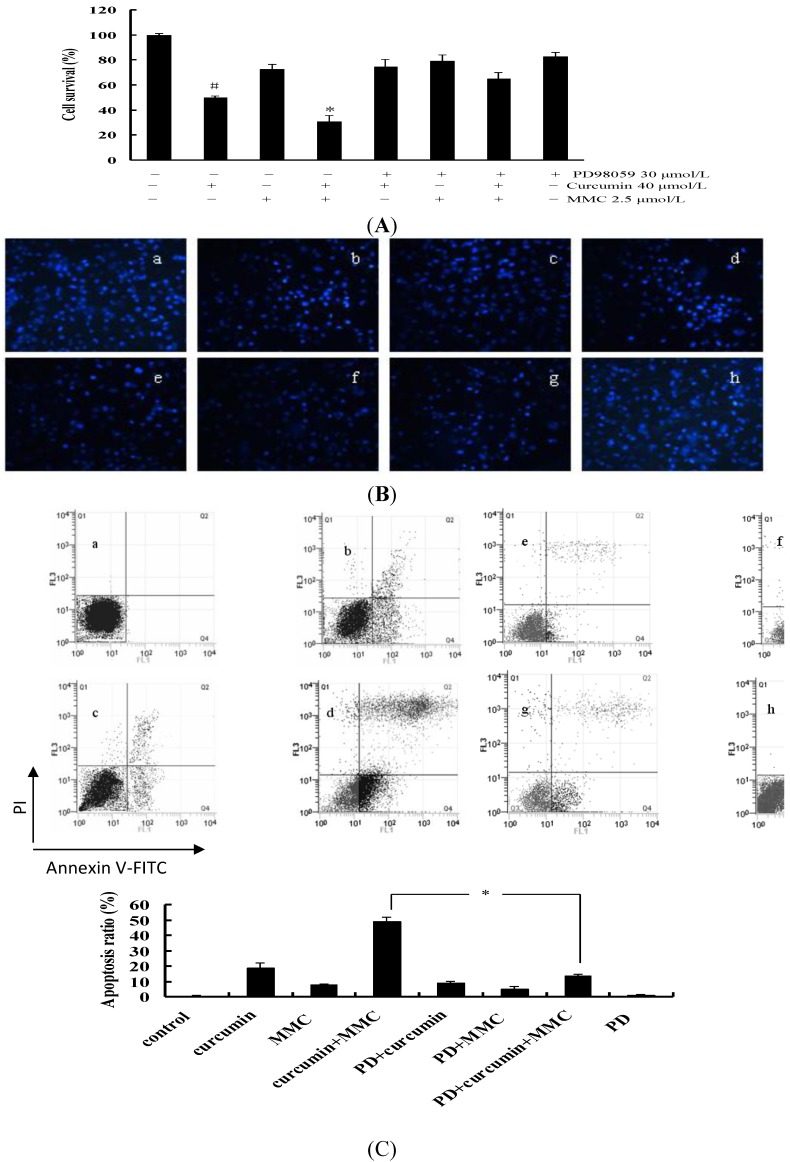
Effect of ERK-specific inhibitor PD98059 alone or PD98059 coupled with different drugs on cell proliferation and apoptosis in MCF-7 cells. (**A**) MCF-7 cells were pretreated with or without 30 μmol/L PD98059 for 2 h before the cells were treated with both 40 μmol/L curcumin and 2.5 μmol/L MMC alone or combination for 48 h. MTT assay was performed as the description in Materials and Methods. All data were expressed as Mean ± SD from three independent experiments. * *p* < 0.05 *versus* curcumin/MMC coupled with PD98059. # *p* < 0.05 *versus* the combinatorial treatment of curcumin and MMC; (**B**) Cell morphology was observed by Hoechst 33258 staining, and then observed under fluorescence microscope (×200); and (**C**) the cells were subjected to the subsequent staining of Annexin V-PI and flow cytometric analysis. Data were expressed as Mean ± SE (*n* = 3). * *p* < 0.05. (**a**) untreated control; (**b**) 40 μmol/L curcumin alone; (**c**) 2.5 μmol/L MMC alone; (**d**), curcumin/MMC combination; (**e**) 30 μmol/L PD98059 combined with 40 μmol/L curcumin; (**f**) 30 μmol/L PD98059 combined with 2.5 μmol/LMMC; (**g**) curcumin/MMC combination coupled with 30 μmol/L PD98059; (**h**) 30 μmol/L PD98059 alone.

In order to assess the role of ERK in the combinatorial treatment, MCF-7 cells were stimulated with ERK-specific inhibitor PD98059 alone or PD98059 coupled with different drugs. As shown in Figure 7A, the rate of cell survival was decreased to 30% due to the combinatorial treatment by MMC and curcumin when compared with the untreated control. However, in the presence of PD98059, cell survival rate revealed an increase by approximately two-fold when compared with the combinatorial treatment without PD98059. Meanwhile, a significant difference between curcumin/MMC coupled with or without PD98059 was observed (*p* < 0.05). This inhibitory effect was reversed by pretreatment with PD98059.

In order to determine the effect of curcumin/MMC treatment on cell apoptosis through ERK pathway, we detected the apoptosis of MCF-7 cells by both flow cytometric and Hoechst 33258 staining analysis. Significant apoptosis was observed in the cells treated with curcumin/MMC combination, while the degree of apoptosis was greatly decreased in the cells treated with curcumin/MMC coupled with PD98059 (Figure 7B).

In order to quantitate the number of apoptotic cells, we performed Annexin V flow cytometry assay on MCF-7 cells exposed to either curcumin/MMC combination or combinatorial treatment coupled with PD98059. The apoptosis rate in the cells treated with curcumin/MMC combination was 49.23%. In the presence of PD98059, the apoptosis rate was reduced to 13.66% (Figure 7C). There was significant difference between them (*p* < 0.05).

The apoptosis rate was consistent with the inhibition of cell growth, which suggested that apoptosis induced by the individual or combinatorial treatment is dependent on the ERK pathway in MCF-7 cells.

MMC, a broad-spectrum chemotherapeutic agent, can inhibit the growth of tumors [26]. In breast cancer treatments, MMC is a third-line chemotherapeutic agent [27] due to its resistance [28], and its application is often accompanied with severe side effects such as renal toxicity [11,29]. Therefore, MMC offers no firm expectation for breast cancer treatment. Recently, the chemoprevention agent known as curcumin (diferuloylmethane) has gained extreme attention because of its relative low toxicity [30] and exhibits anti-cancer activity both *in vitro* and *in vivo* through a variety of mechanisms [31]. We have found that the combinatorial treatment of curcumin and MMC can significantly inhibit the cell growth of breast cancer [11,12]. In order to explore the synergistic mechanisms of curcumin combined with MMC, we have screened the associated genes by cDNA microarray analysis.

The cDNA microarray technology can provide the expression profile of all genes simultaneously, and is an important tool in the dissection of signal transduction pathways. In the present study, curcumin synergistically enhanced the anti-tumor capability of MMC at the dose of 1.5 mg/kg to reduce tumor mass from 64.5% to 86.5% (Figure 1). Tumor tissues administrated with curcumin combined with MMC at the doses of 1.0 and 1.5 mg/kg were examined by cDNA microarray analysis. Curcumin/MMC at the dose of 1.5 mg/kg induced 1501 DE genes and 25 GO terms when compared with curcumin/MMC at the dose of 1.0 mg/kg (Figure 2). Moreover, the apoptosis and cell cycle were mainly related to the synergistic combination of curcumin and MMC in signaling pathway analysis.

In order to clarify the relationship among these genes, Signal–Net network analysis was used to explore these relationships among different treatments. The considered evidence is the source of the interaction database from KEGG. Networks are stored and presented as graphs, where the nodes are mainly genes or proteins, and edges represent the related types between the nodes. Previous reports have shown that the activation of MEK by mitogenic stimuli contributes to the differentiation, proliferation, and survival of cells [32]. Some responsiveness is regulated by ERK and MAPK in breast cancer cells. From this network, two genes associated with apoptosis, ERK and p38 may be the mainly related genes (Figure 4 and Table 1). Notably, these results suggest that the synergistic combinatorial treatment-induced apoptosis may be involved in ERK and p38 pathways in breast cancer cells.

It has been reported that cell survival and apoptosis are regulated through ERK/MAPK in various cancer cells such as human pancreatic cancer cells [33,34,35] and human endometrial cancer cells [36]. Moreover, ursodeoxycholic acid (UDCA) enhanced the phosphorylation of ERK1/2 and MEK1/2. The prevention of MEK by the pharmacologic inhibitors PD98059 and U0126, resulted in the decrease of UDCA-induced apoptosis, as shown by the reduction of apoptotic body formation, caspase-8 activity, and caspase-3, -6 and PARP cleavage, indicating that ERK exerts pro-apoptotic activity upon exposure to UDCA [37]. In order to validate whether the apoptosis induced by combinatorial treatment was regulated by ERK, MEK-specific inhibitor PD98059 was used. As shown in Figure 7, cell survival was decreased and apoptosis was increased by curcumin combined with MMC treatment. However, these effects were rescued by PD98059. In this study, ERK signaling is involved in the inhibition of cell growth and apoptosis induction by combinatorial treatment in MCF-7 cells.

qRT-PCR and Western blot are used to validate differential expression of ERK-associated genes and proteins have been carried out by comparing curcumin and MMC-treated samples with control samples (Figure 5). The combination of curcumin and MMC 1.5 mg/kg increased the expression level of ERK mRNA and protein.

In order to determine the effect of curcumin/MMC treatment on other breast cancer cell lines, we detected cell survival of MDA-MB-231 cells by performing MTT assay. Curcumin reduced the concentration required for MMC to suppress the growth of breast cancer MDA-MB-231 cells (Figure 6). Therefore, different breast cancer cell types were equally sensitive to this treatment.

## 3. Experimental Section

### 3.1. Reagents and Cell Culture

MMC was purchased from International Chemical and Nuclear Corporation (Hamilton, ON, Canada). Curcumin was obtained from National Institute for the Control of Pharmaceutical and Biological Products (Beijing, China). PD98059 was ordered from Biomol (Plymouth Meeting, PA, USA). The antibodies against p-ERK, ERK, p-p38 and p38 were obtained from Cell Signaling Technology (Danvers, MA, USA). Hoechst 33258 was purchased from Invitrogen (Carlsbad, CA, USA). Human breast cancer MCF-7 cells were purchased from the American Type Culture Collection (ATCC) (Manassas, VA, USA) and cultured in RPMI 1640 medium supplemented with 10% fetal calf serum (FBS) and 0.01 mg/mL insulin at 37 °C with 5% CO_2_ in a humidified atmosphere.

### 3.2. Tumor Xenograft and Treatment

Female *nu*/*nu* athymic mice (7 weeks of age) were obtained from Academia Sinica (Shanghai, China). MCF-7 cells (1 × 10^7^/mL) were inoculated into the mammary fat pad (m.f.p.) of mice and 17β-estradiol was intraperitoneally injected before inoculation [38,39]. Once palpable tumors were developed (approximately 2 weeks), the mice were randomly divided into 9 groups (*n* = 8). Treatment groups included curcumin (100 mg/kg) [11], MMC (1.0, 1.5 and 2 mg/kg) and combinatorial treatment of curcumin (100 mg/kg) and MMC (1.0, 1.5 and 2 mg/kg) groups. All drugs were administrated intraperitoneally. Untreated groups were divided into normal and control groups with the injection of physiological saline. After 4 weeks of treatment, the mice were sacrificed followed by tumor removal in trizol. All procedures were conformed to the consideration of animal welfare and approved by the ethical committee (09001, 5 March 2014) of Shanghai Traditional Chinese Medicine.

### 3.3. Microarray Detection

Total RNAs of tumor tissues from different treatments using curcumin/MMC at the doses of 1.0 and 1.5 mg/kg and saline were extracted using TRIzol (Invitrogen) and the RNeasy kit (Qiagen, Hilden, Germany) according to manufacturer’s instructions including a DNase digestion step. RNA concentration was evaluated on the Nanodrop ND-1000 and analyzed by denaturing gel electrophoresis. The samples were amplified and labeled using a NimbleGen One-Color DNA Labeling Kit (Roch, Basel, Switzerland) and hybridized in NimbleGen Hybridization System (Roch). After hybridization and washing, the processed slides were scanned with the Axon GenePix 4000B microarray scanner (Molecular Devices, Sunnyvale, CA, USA). Raw data were extracted as pair files by NimbleScan software (version 2.5) (Roch).

### 3.4. Data Analysis

In order to evaluate the effects of different treatments, we compared the mRNA expression of tumor tissues subjected to the treatments of curcumin/MMC at the doses of 1.0 and 1.5 mg/kg, and the control, respectively. The relative mRNA expression levels were further normalized utilizing the median over all samples. The weighted differentially expressed (DE) genes were calculated using the random variance model *t*-test. A significant difference was considered at the fold change larger than 1.5 and *p* < 0.05. Among these inferred DE genes, the enrichment was analyzed using ingenuity pathway analysis (IPA), and significance analysis was determined when *p* values were corrected for the false discovery rate (FDR). Gene sets containing less than 5 overlapped genes were removed from the DAVID analysis. In our analysis, gene ontology (GO) terms and pathways with an FDR-adjusted *p* value of less than 0.05 were retained.

### 3.5. Regulatory Network Construction of Weighted Genes

Direct binding between different genes can also reveal shared functionality. We built the weighted gene regulatory networks for DE genes using Signal–Net [40,41], which is a network-building method based on KEGG interaction database. In the weighted regulatory network, the nodes represent genes, and the edges represent the connection strength (adjacency). As an example, if two genes interact with each other, an interaction edge is assigned between two genes where arrows or smooth edges indicate the activation or inhibition.

### 3.6. Quantitative RT-PCR (qRT-PCR)

The gene expression levels in cDNA microarray analysis were validated by qRT-PCR. The sequence of MAPK1 (ERK) oligonucleotide is 5'-CAGGTGTTCGACGTAGGGC-3'. Total RNA (1 µg) was employed to prepare cDNA via reverse transcription. All reactions were performed in a final volume of 20 μL. qRT-PCR reaction conditions were as follows: activation at 95 °C for 10 min with 40 cycles of denaturation at 95 °C for 15 s, primer annealing and extension at 60 °C for 1 min and ramping back to 95 °C. Melting curve analysis of all samples was routinely performed to ascertain that only the expected products were generated. A fluorescence reading was determined the extent of amplification at the end of each cycle. The expression levels of target genes were normalized to the expression of GAPDH. The qRT-PCR for each gene was determined in duplicate, and each experiment was repeated three times.

### 3.7. Western Blot Analysis

Tumor tissues were homogenized and lysed in lysis buffer containing 2 M sodium chloride, 10% NP-40, 10% SDS, 1 M Tris–HCl, 1 g/L phenyl-methylsulphonyl fluoride (PMSF), 0.1 g/L aprotinin and 0.01 g/L leupeptin. The cell lysates were subjected to SDS-PAGE and then blotted onto PVDF membrane. After membrane was blocked with BSA for 1 h, the expression of proteins was detected using primary antibodies (1:1000) and secondary antibodies (1:800) conjugated with horseradish peroxidase and enhanced chemiluminescence (ECL) reagents (Pharmacia, Buckinghamshire, UK). Quantitative analysis of Western blots was carried out using Alpha Ease FC (FluorChem FC2) software (Santa Clara, CA, USA). The density ratio of proteins to GAPDH as spot density was calculated using the analysis tools.

### 3.8. Cell Growth Inhibition Test

The inhibition of cell growth was determined by 3-(4,5-dimethylthiazol-2-yl)-2,5-diphenyltetrazolium bromide (MTT) assay. Fifteen various concentrations of MMC, with or without curcumin, were then added to MCF-7 or MDA-MB-231 cells for varying length of times, followed by the addition of MTT for another 4 h. After removal of culture solution, the remaining MTT formazan crystals were dissolved with DMSO and measured at 490 nm with a microplate reader. The percentage of inhibition was calculated as followed by the formula: Inhibition ratio (IR) (%) = (1 − OD_sample_/OD_control_) × 100%.

### 3.9. Flow Cytometric Analysis

MCF-7 cells (10^6^/mL) were cultured in 6-well plates, and reached up to 70%–80% confluence after seeding cells for 6 h. The media containing FCS were not changed, and then treated with 2.5 μmol/L MMC, 40 μmol/L curcumin and 30 μmol/L PD98059 alone or combination. Cells were subjected to annexin V-PI dual staining assay according to the manufacturer’s protocol. Stained cells were analyzed by fluorescence activating cell sorter (FACS) (Becton Dickinson, San Jose, CA, USA) and the apoptotic cell population was determined using ModFit LT 3.0 software (Becton Dickinson).

### 3.10. Hoechst 33258 Staining

Cells were seeded on the slides at a density of 5 × 10^4^/mL in 6-well plates. After treatment as mentioned above, the cells from all five groups were washed twice with phosphate-buffered saline (PBS), fixed in 4% paraformaldehyde for 10 min, and then stained with Hoechst 33258 for 5 min. After the cells were washed twice with PBS, the cells were observed under a fluorescence microscope. The nuclei of living cells were homogeneous blue, but the nuclei of apoptotic cells were compact, condensed and whitish blue.

## 4. Conclusions

In summary, the combination of curcumin and MMC 1.5 mg/kg can synergistically inhibit tumor growth in MCF-7 breast cancer xenografts and induce apoptosis in breast cancer MCF-7 cells through ERK pathway.
